# Draft Genome Sequence of *Bacillus cecembensis* PN5^T^ (DSM 21993), a Psychrotolerant Bacterium Isolated from Soil Samples near the Pindari Glacier

**DOI:** 10.1128/genomeA.01687-15

**Published:** 2016-02-04

**Authors:** Jie-ping Wang, Bo Liu, Guo-hong Liu, Ci-bin Ge, Qian-qian Chen, Jian-mei Che, De-ju Chen

**Affiliations:** Agricultural Bio-Resources Research Institute, Fujian Academy of Agricultural Sciences, Fuzhou, Fujian, China

## Abstract

*Bacillus cecembensis* PN5^T^ is a Gram-positive, aerobic, and spore-forming bacterium with very high intrinsic heat resistance. Here, we report the 4.72-Mb draft genome sequence of *B. cecembensis* PN5^T^, the first genome sequence of this species, which will promote its fundamental research.

## GENOME ANNOUNCEMENT

The type strain PN5^T^ (= LMG 23935^T^ = MTCC9127^T^ = CM 15113^T^) of *Bacillus cecembensis* was isolated from soil samples collected at an altitude of approximately 3,500 m near the Pindari Glacier of the Indian Himalayas (1). It is one of the very few psychrotolerant species of the genus *Bacillus* (1). It appears to have a close relationship with *Bacillus silvestris* (now *Solibacillus silvestris*), with a sequence similarity of 97.2% (1, 2). Except for the taxonomic literature, no additional information for *B. cecembensis* has been obtained so far. Because there is no available genomic information for *B. cecembensis*, its type strain PN5^T^ was selected as one of the research objects in our “genome sequencing project for genomic taxonomy and phylogenomics of *Bacillus*-like bacteria” (J.-P. Wang, B. Liu, G.-H. Liu, J.-M. Che, Zheng Chen, M.-C. Chen, and H. Shi, unpublished data). Here, we present the first draft genome sequence of *B. cecembensis*.

The genome sequence of *B. cecembensis* PN5^T^ was obtained by paired-end sequencing on the Illumina HiSeq 2500 system. One DNA library with an insert size of 252 bp was constructed and sequenced. After filtering of the 2.16 Gb of raw data, 2.04 Gb of clean data were obtained, providing approximately 408-fold coverage. The reads were assembled via the SOAP*denovo* software version 1.05 (3), using a key parameter K setting of 76. Through the data assembly, 88 scaffolds with a total length of 4,723,910 bp were obtained, and the scaffold *N*_50_ was 174,129 bp. The average length of the scaffolds was 53,681 bp, and the longest and shortest scaffolds were 623,960 bp and 523 bp, respectively. A total 94.44% clean reads were aligned back to the genome, which covered 99.33% of the sequence.

The annotation of the genome was performed using the NCBI Prokaryotic Genome Annotation Pipeline (PGAP) (http://www.ncbi.nlm.nih.gov/genomes/static/Pipeline.html) utilizing the GeneMark, Glimmer, and tRNAscan-SE tools (4). A total of 4,727 genes were predicted, including 4,676 coding sequences (CDSs), 47 tRNAs, and 4 rRNA genes. There were 3,203 and 1,947 genes assigned to COG and KEGG databases, respectively. The average DNA G+C content was 36.84%, agreeing with the value 43.7 mol% (1).

### Nucleotide sequence accession numbers.

This whole-genome shotgun project has been deposited at DDBJ/EMBL/GenBank under the accession number LMBZ00000000. The version described in this paper is version LMBZ01000000.
